# (*E*)-3-(4-Fluoro­phen­yl)-1-phenyl-2-propen-1-one

**DOI:** 10.1107/S1600536809037635

**Published:** 2009-09-26

**Authors:** Lin-Hai Jing

**Affiliations:** a School of Chemistry and Chemical Engineering, China West Normal University, Nanchong 637002, People’s Republic of China

## Abstract

In the title compound, C_15_H_11_FO, the configuration of the keto group with respect to the olefinic double bond is *s–cis*. The dihedral angle between the planes of the two benzene rings is 10.61 (10)°. The crystal packing is stabilized by C—H⋯π inter­actions involving both benzene rings.

## Related literature

For the synthesis, see: Chimenti *et al.* (2008 ▶). For the biological activity of chalcone derivatives, see: Dimmock *et al.* (1999 ▶).
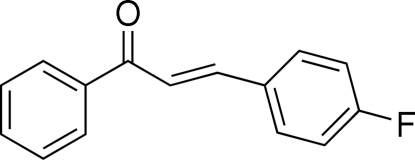


## Experimental

### 

#### Crystal data

C_15_H_11_FO
*M* *_r_* = 226.24
Monoclinic, 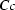

*a* = 24.926 (9) Å
*b* = 5.6940 (19) Å
*c* = 7.749 (3) Å
β = 94.747 (5)°
*V* = 1096.0 (6) Å^3^
*Z* = 4
Mo *K*α radiation
μ = 0.10 mm^−1^
*T* = 93 K
0.40 × 0.33 × 0.30 mm

#### Data collection

Rigaku SPIDER diffractometer
Absorption correction: none
4214 measured reflections
1256 independent reflections
1174 reflections with *I* > 2σ(*I*)
*R*_int_ = 0.027

#### Refinement

*R*[*F* ^2^ > 2σ(*F* ^2^)] = 0.032
*wR*(*F* ^2^) = 0.066
*S* = 1.05
1256 reflections
154 parameters
2 restraints
H-atom parameters constrained
Δρ_max_ = 0.20 e Å^−3^
Δρ_min_ = −0.14 e Å^−3^

### 

Data collection: *RAPID-AUTO* (Rigaku/MSC, 2004 ▶); cell refinement: *RAPID-AUTO*; data reduction: *RAPID-AUTO*; program(s) used to solve structure: *SHELXS97* (Sheldrick, 2008 ▶); program(s) used to refine structure: *SHELXL97* (Sheldrick, 2008 ▶); molecular graphics: *XP* in *SHELXTL* (Sheldrick, 2008 ▶); software used to prepare material for publication: *SHELXL97*.

## Supplementary Material

Crystal structure: contains datablocks global, I. DOI: 10.1107/S1600536809037635/ci2914sup1.cif

Structure factors: contains datablocks I. DOI: 10.1107/S1600536809037635/ci2914Isup2.hkl

Additional supplementary materials:  crystallographic information; 3D view; checkCIF report

## Figures and Tables

**Table 1:** Hydrogen-bond geometry (Å, °)

*D*—H⋯*A*	*D*—H	H⋯*A*	*D*⋯*A*	*D*—H⋯*A*
C1—H1⋯*Cg*1^i^	0.95	2.89	3.592 (3)	132
C4—H4⋯*Cg*1^ii^	0.95	2.93	3.646 (6)	133
C12—H12⋯*Cg*2^iii^	0.95	2.85	3.505 (8)	127

Symmetry codes: (i) 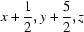; (ii) 
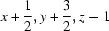
; (iii) 
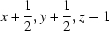
. *Cg*1 and *Cg*2 are the centroids of the C1–C6 and C10–C15 rings, respectively.

**Fig. 1.**
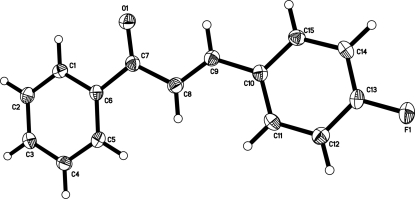
The molecular structure of the title compound, showing 50% probability displacement ellipsoids and the atomic numbering.

## supplementary crystallographic information

### Comment

Chalcone derivatives are a class of important compounds that possess antiprotzoal, antihelmintic, amoebicidal, anti-ulcer, antiviral, insecticidal, antibacterial, anticancer, cytotoxic and immunosuppressive activities (Dimmock *et al.*, 1999). The author reports here the crystal structure of the title compound, a chalocone derivative.

Bond lengths and angles in the title molecule (Fig.1) are normal. The configuration of the keto group with respect to the olefinic double bond is s-*cis*, with a O1—C7—C8—C9 torsion angle of -7.1 (3)°. The C1-C6 and C10-C15 benzene rings form a dihedral angle of 10.61 (10)°.

The crystal packing is stabilized by C—H···π interactions involving both benzene rings (Table 1; Cg1 and Cg2 are centroids of the C1-C6 and C10-C15 rings, respectively).

### Experimental

The title compound was synthesized according to the method reported in the literature (Chimenti *et al.*, 2008). Colourless single crystals suitable for X-ray diffraction were obtained by slow evaporation of a acetone solution of the compound.

### Refinement

H atoms were placed in calculated positions, with C-H = 0.95 Å, and refined using a riding model, with *U*_iso_(H) = 1.2*U*_eq_(C). In the absence of significant anomalous scattering effects, Friedel pairs were averaged.

### Crystal data

**Table:**

C_15_H_11_FO	*F*(000) = 472
*M**_r_* = 226.24	*D*_x_ = 1.371 Mg m^−^^3^
Monoclinic, *C**c*	Mo *K*α radiation, λ = 0.71073 Å
Hall symbol: C -2yc	Cell parameters from 1803 reflections
*a* = 24.926 (9) Å	θ = 3.2–27.5°
*b* = 5.6940 (19) Å	µ = 0.10 mm^−^^1^
*c* = 7.749 (3) Å	*T* = 93 K
β = 94.747 (5)°	Block, colourless
*V* = 1096.0 (6) Å^3^	0.40 × 0.33 × 0.30 mm
*Z* = 4	

### Data collection

**Table:**

Rigaku SPIDER diffractometer	1174 reflections with *I* > 2σ(*I*)
Radiation source: Rotating anode	*R*_int_ = 0.027
graphite	θ_max_ = 27.5°, θ_min_ = 3.3°
ω scans	*h* = −32→32
4214 measured reflections	*k* = −7→6
1256 independent reflections	*l* = −10→9

### Refinement

**Table:**

Refinement on *F*^2^	Primary atom site location: structure-invariant direct methods
Least-squares matrix: full	Secondary atom site location: difference Fourier map
*R*[*F*^2^ > 2σ(*F*^2^)] = 0.032	Hydrogen site location: inferred from neighbouring sites
*wR*(*F*^2^) = 0.066	H-atom parameters constrained
*S* = 1.05	*w* = 1/[σ^2^(*F*_o_^2^) + (0.02*P*)^2^ + 0.6*P*] where *P* = (*F*_o_^2^ + 2*F*_c_^2^)/3
1256 reflections	(Δ/σ)_max_ = 0.010
154 parameters	Δρ_max_ = 0.20 e Å^−^^3^
2 restraints	Δρ_min_ = −0.14 e Å^−^^3^

### Special details

**Table:**

Geometry. All e.s.d.'s (except the e.s.d. in the dihedral angle between two l.s. planes) are estimated using the full covariance matrix. The cell e.s.d.'s are taken into account individually in the estimation of e.s.d.'s in distances, angles and torsion angles; correlations between e.s.d.'s in cell parameters are only used when they are defined by crystal symmetry. An approximate (isotropic) treatment of cell e.s.d.'s is used for estimating e.s.d.'s involving l.s. planes.
Refinement. Refinement of *F*^2^ against ALL reflections. The weighted *R*-factor *wR* and goodness of fit *S* are based on *F*^2^, conventional *R*-factors *R* are based on *F*, with *F* set to zero for negative *F*^2^. The threshold expression of *F*^2^ > σ(*F*^2^) is used only for calculating *R*-factors(gt) *etc*. and is not relevant to the choice of reflections for refinement. *R*-factors based on *F*^2^ are statistically about twice as large as those based on *F*, and *R*- factors based on ALL data will be even larger.

### Fractional atomic coordinates and isotropic or equivalent isotropic displacement parameters (Å^2^)

**Table:**

	*x*	*y*	*z*	*U*_iso_*/*U*_eq_	
F1	0.78252 (5)	−0.0769 (2)	0.41272 (15)	0.0283 (3)	
O1	0.54146 (6)	0.8936 (3)	0.5750 (2)	0.0298 (4)	
C1	0.43870 (9)	0.9349 (4)	0.4181 (3)	0.0201 (5)	
H1	0.4526	1.0584	0.4912	0.024*	
C2	0.38614 (9)	0.9458 (4)	0.3436 (3)	0.0224 (5)	
H2	0.3642	1.0767	0.3660	0.027*	
C3	0.36564 (8)	0.7667 (4)	0.2369 (3)	0.0220 (5)	
H3	0.3296	0.7749	0.1868	0.026*	
C4	0.39742 (9)	0.5758 (4)	0.2026 (3)	0.0222 (5)	
H4	0.3834	0.4539	0.1282	0.027*	
C5	0.45022 (9)	0.5632 (4)	0.2780 (3)	0.0205 (5)	
H5	0.4720	0.4315	0.2555	0.025*	
C6	0.47111 (8)	0.7420 (4)	0.3855 (3)	0.0180 (4)	
C7	0.52705 (8)	0.7380 (4)	0.4726 (3)	0.0205 (4)	
C8	0.56390 (9)	0.5435 (4)	0.4363 (3)	0.0217 (5)	
H8	0.5512	0.4142	0.3669	0.026*	
C9	0.61504 (8)	0.5513 (4)	0.5016 (3)	0.0199 (4)	
H9	0.6250	0.6836	0.5718	0.024*	
C10	0.65773 (9)	0.3806 (4)	0.4783 (3)	0.0185 (4)	
C11	0.64836 (9)	0.1657 (4)	0.3938 (3)	0.0214 (5)	
H11	0.6127	0.1239	0.3526	0.026*	
C12	0.69023 (9)	0.0130 (4)	0.3692 (3)	0.0225 (5)	
H12	0.6838	−0.1310	0.3091	0.027*	
C13	0.74170 (9)	0.0754 (4)	0.4343 (3)	0.0211 (5)	
C14	0.75290 (8)	0.2834 (4)	0.5203 (3)	0.0212 (4)	
H14	0.7886	0.3216	0.5635	0.025*	
C15	0.71063 (9)	0.4355 (4)	0.5420 (3)	0.0207 (4)	
H15	0.7176	0.5799	0.6012	0.025*	

### Atomic displacement parameters (Å^2^)

**Table:**

	*U*^11^	*U*^22^	*U*^33^	*U*^12^	*U*^13^	*U*^23^
F1	0.0222 (7)	0.0284 (7)	0.0342 (8)	0.0079 (6)	0.0022 (5)	0.0009 (6)
O1	0.0228 (8)	0.0330 (9)	0.0326 (9)	0.0047 (7)	−0.0034 (7)	−0.0121 (8)
C1	0.0228 (11)	0.0192 (11)	0.0185 (11)	−0.0002 (9)	0.0021 (8)	−0.0011 (9)
C2	0.0221 (11)	0.0227 (11)	0.0227 (11)	0.0043 (9)	0.0032 (9)	0.0017 (9)
C3	0.0177 (10)	0.0265 (11)	0.0218 (11)	0.0006 (9)	0.0017 (9)	0.0031 (9)
C4	0.0218 (11)	0.0232 (11)	0.0215 (11)	−0.0036 (9)	0.0012 (8)	−0.0011 (9)
C5	0.0218 (11)	0.0205 (11)	0.0196 (10)	0.0027 (9)	0.0046 (9)	0.0002 (9)
C6	0.0177 (10)	0.0206 (11)	0.0161 (10)	0.0007 (8)	0.0031 (8)	0.0028 (8)
C7	0.0187 (10)	0.0230 (11)	0.0199 (10)	0.0001 (9)	0.0026 (8)	0.0005 (9)
C8	0.0228 (10)	0.0215 (11)	0.0206 (10)	0.0014 (9)	0.0012 (8)	−0.0019 (9)
C9	0.0209 (10)	0.0209 (11)	0.0178 (10)	0.0023 (9)	0.0012 (8)	−0.0003 (9)
C10	0.0179 (10)	0.0210 (10)	0.0163 (10)	0.0021 (9)	0.0004 (8)	0.0033 (9)
C11	0.0177 (10)	0.0249 (11)	0.0211 (11)	−0.0018 (9)	−0.0013 (8)	0.0021 (9)
C12	0.0247 (11)	0.0222 (11)	0.0206 (11)	0.0014 (9)	0.0012 (9)	0.0005 (9)
C13	0.0191 (11)	0.0229 (11)	0.0217 (12)	0.0047 (9)	0.0040 (8)	0.0042 (9)
C14	0.0155 (10)	0.0259 (11)	0.0218 (10)	−0.0017 (9)	−0.0005 (8)	0.0030 (10)
C15	0.0214 (11)	0.0204 (10)	0.0199 (11)	−0.0005 (9)	−0.0009 (8)	0.0004 (9)

### Geometric parameters (Å, °)

**Table:**

F1—C13	1.358 (2)	C8—C9	1.333 (3)
O1—C7	1.223 (3)	C8—H8	0.95
C1—C2	1.389 (3)	C9—C10	1.463 (3)
C1—C6	1.399 (3)	C9—H9	0.95
C1—H1	0.95	C10—C11	1.398 (3)
C2—C3	1.384 (3)	C10—C15	1.404 (3)
C2—H2	0.95	C11—C12	1.383 (3)
C3—C4	1.384 (3)	C11—H11	0.95
C3—H3	0.95	C12—C13	1.386 (3)
C4—C5	1.397 (3)	C12—H12	0.95
C4—H4	0.95	C13—C14	1.376 (3)
C5—C6	1.389 (3)	C14—C15	1.385 (3)
C5—H5	0.95	C14—H14	0.95
C6—C7	1.498 (3)	C15—H15	0.95
C7—C8	1.481 (3)		
			
C2—C1—C6	119.9 (2)	C7—C8—H8	120.3
C2—C1—H1	120.0	C8—C9—C10	127.8 (2)
C6—C1—H1	120.0	C8—C9—H9	116.1
C3—C2—C1	120.3 (2)	C10—C9—H9	116.1
C3—C2—H2	119.8	C11—C10—C15	118.37 (19)
C1—C2—H2	119.8	C11—C10—C9	122.95 (19)
C2—C3—C4	120.3 (2)	C15—C10—C9	118.68 (19)
C2—C3—H3	119.8	C12—C11—C10	121.0 (2)
C4—C3—H3	119.8	C12—C11—H11	119.5
C3—C4—C5	119.6 (2)	C10—C11—H11	119.5
C3—C4—H4	120.2	C11—C12—C13	118.4 (2)
C5—C4—H4	120.2	C11—C12—H12	120.8
C6—C5—C4	120.43 (19)	C13—C12—H12	120.8
C6—C5—H5	119.8	F1—C13—C14	119.03 (19)
C4—C5—H5	119.8	F1—C13—C12	118.19 (19)
C5—C6—C1	119.37 (19)	C14—C13—C12	122.8 (2)
C5—C6—C7	123.20 (18)	C13—C14—C15	118.11 (19)
C1—C6—C7	117.42 (18)	C13—C14—H14	120.9
O1—C7—C8	120.79 (19)	C15—C14—H14	120.9
O1—C7—C6	119.56 (19)	C14—C15—C10	121.3 (2)
C8—C7—C6	119.64 (18)	C14—C15—H15	119.3
C9—C8—C7	119.4 (2)	C10—C15—H15	119.3
C9—C8—H8	120.3		
			
C6—C1—C2—C3	−0.1 (3)	C7—C8—C9—C10	−178.7 (2)
C1—C2—C3—C4	−0.3 (3)	C8—C9—C10—C11	−6.7 (3)
C2—C3—C4—C5	0.7 (3)	C8—C9—C10—C15	172.6 (2)
C3—C4—C5—C6	−0.6 (3)	C15—C10—C11—C12	−1.7 (3)
C4—C5—C6—C1	0.2 (3)	C9—C10—C11—C12	177.7 (2)
C4—C5—C6—C7	179.0 (2)	C10—C11—C12—C13	1.6 (3)
C2—C1—C6—C5	0.2 (3)	C11—C12—C13—F1	178.78 (19)
C2—C1—C6—C7	−178.7 (2)	C11—C12—C13—C14	−0.8 (3)
C5—C6—C7—O1	−175.0 (2)	F1—C13—C14—C15	−179.54 (18)
C1—C6—C7—O1	3.9 (3)	C12—C13—C14—C15	0.0 (3)
C5—C6—C7—C8	4.0 (3)	C13—C14—C15—C10	−0.1 (3)
C1—C6—C7—C8	−177.2 (2)	C11—C10—C15—C14	0.9 (3)
O1—C7—C8—C9	−7.1 (3)	C9—C10—C15—C14	−178.5 (2)
C6—C7—C8—C9	174.0 (2)		

### Hydrogen-bond geometry (Å, °)

**Table:**

*D*—H···*A*	*D*—H	H···*A*	*D*···*A*	*D*—H···*A*
C1—H1···Cg1^i^	0.95	2.89	3.592 (3)	132
C4—H4···Cg1^ii^	0.95	2.93	3.646 (6)	133
C12—H12···Cg2^iii^	0.95	2.85	3.505 (8)	127

Symmetry codes: (i) *x*+1/2, *y*+5/2, *z*; (ii) *x*+1/2, *y*+3/2, *z*−1; (iii) *x*+1/2, *y*+1/2, *z*−1.
